# Prognostic relevance of lymphocytopenia, monocytopenia and lymphocyte-to-monocyte ratio in primary myelodysplastic syndromes: a single center experience in 889 patients

**DOI:** 10.1038/bcj.2017.30

**Published:** 2017-03-31

**Authors:** L Saeed, M M Patnaik, K H Begna, A Al-Kali, M R Litzow, C A Hanson, R P Ketterling, L F Porrata, A Pardanani, N Gangat, A Tefferi

**Affiliations:** 1 Department of Medicine, Division of Hematology, Mayo Clinic, Rochester, MN, USA; 2 Department of Laboratory Medicine, Division of Hematopathology, Mayo Clinic, Rochester, MN, USA; 3 Department of Laboratory Medicine, Division of Cytogenetics, Mayo Clinic, Rochester, MN, USA

## Abstract

Current prognostic models for myelodysplastic syndromes (MDS), including the Revised International Prognostic Scoring System (IPSS-R), do not account for host immunity. We retrospectively examined the prognostic relevance of monocytopenia, lymphocytopenia and lymphocyte-to-monocyte ratio (LMR) in a cohort of 889 patients with primary MDS. After a median follow-up of 27 months, 712 (80%) deaths and 116 (13%) leukemic transformation were documented. In univariate analysis, subnormal absolute lymphocyte count (ALC) <0.9 × 10^9^/l; *P*=0.001), ALC<1.2 × 10^9^/l (*P*=0.0002), subnormal absolute monocyte count (AMC) <0.3 × 10^9^/l (*P*=0.0003), LMR (*P*⩽0.0001) and LMR⩾5 (*P*=0.03) were all associated with inferior overall survival. In multivariable analysis that included other risk factors, significance was retained for LMR (*P*=0.02) and became borderline for ALC <1.2 × 10^9^/l (*P*=0.06). Analysis in the context of IPSS-R resulted in *P*-values of 0.06 for ALC<1.2 × 10^9^/l, 0.7 for monocytopenia and 0.2 for LMR. Leukemia-free survival was not affected by ALC, AMC or LMR. The observations from the current study suggest a possible detrimental role for altered host immunity in primary MDS, which might partly explain the therapeutic benefit of immune-directed therapy, including the use of immune modulators; however, IPSS-R-independent prognostic value for either ALC or AMC was limited.

## Introduction

Myelodysplastic syndromes (MDS) are a group of heterogeneous clonal hematopoietic stem cell disorders with an inherent tendency for leukemic transformation.^1^ MDS is characterized by ineffective hematopoiesis, manifested by morphological dysplasia in the bone marrow and by peripheral blood cytopenia(s).^2^ The pathophysiology of the disease remains largely elusive. In order to have accurate risk stratification of patients with primary MDS, formal prognostic models have been developed over the years. The International Prognostic Scoring System (IPSS) was introduced in 1997 followed by the World Health Organization Prognostic Scoring System in 2007, the global MD Anderson score in 2008 and the most recent Revised International Prognostic Scoring System (IPSS-R) in 2012. These prognostic models for MDS consider the number and severity of cytopenias, including anemia, thrombocytopenia and neutropenia, need for red blood cell transfusions, karyotype, bone marrow and peripheral blood blast percentage, leukocytosis, morphological subtypes, age and performance status.^3, 4, 5, 6^

Dysregulation of host immunity is considered to be critical in the pathogenesis and progression of primary MDS.^7, 8^ In general, early lymphocyte recovery after chemotherapy or after stem cell transplant has been shown to be associated with superior survival in various hematological and non-hematological malignancies.^9, 10, 11, 12^ In the context of primary MDS, prior studies have shown that the French–American–British morphological classification, neutrophil count and CD8+ T-lymphocyte count had the best discriminatory capacity for predicting survival <1 year and French–American–British classification, neutrophil count and serum tumor necrosis factor levels best predicted conversion to acute leukemia.^13^ Our previous studies have shown absolute lymphocyte count (ALC) at the time of diagnosis to be an independent prognostic factor for survival in patients with both del (5q)^14^ and non-del (5q) MDS.^8^ The IPSS-R, although a useful prognostic tool, does not consider the prognostic role of lymphocytopenia or monocytopenia. It is possible that these surrogates of host immunity may partly account for disease progression and poor survival; the current study examines the possibility by studying the prognostic significance of ALC, absolute monocyte count (AMC) and lymphocyte-to-monocyte ratio (LMR) at the time of diagnosis in primary MDS, in terms of both overall and leukemia-free survival.

## Materials and Methods

We retrospectively recruited 889 patients with primary MDS who had been untreated at the time of referral to our institution and in whom the laboratory characteristics at the time of diagnosis were recorded. The diagnosis of MDS and leukemic transformation were according to the World Health Organization criteria.^2^ All morphological and cytogenetic assessments had to be either performed or reviewed at our institution for study inclusion. Our institutional normal range was 0.9–2.9 × 10^9^/l for lymphocyte count and 0.3 to 0.9 × 10^9^/l for monocyte count. Follow-up information was updated in January 2015. Approval for the retrospective review of these records was obtained from the Mayo Clinic institutional review board and was in accordance with US federal regulations and the Declaration of Helsinki.

Patients were stratified according to ALC, AMC and LMR. Comparison of survival and other clinical parameters were performed between patients with subnormal (<0.9 × 10^9^/l) and normal ALC: patients with ALC<1.2 × 10^9^/l and ALC (1.2–2.9 × 10^9^/l) in the study cohort, based on our previous observation^8^; patients with and without subnormal AMC (<0.3 × 10^9^/l); and patients with and without LMR⩾5, based on published reports of relevance^15^; the latter study showed that patients with LMR>5 experienced shorter survival with median of 67 vs 126 months. Differences in the distribution of continuous variables between categories were analyzed by either Mann–Whitney (for comparison of two groups) or Kruskal–Wallis test (comparison of three or more groups). Patient groups with nominal variables were compared by χ^2^ test. Overall survival analysis was considered from the date of diagnosis to date of death or last contact. Leukemia-free survival was determined from the time of diagnosis to the time the event occurred after diagnosis. All survival curves were prepared by the Kaplan–Meier method and compared by the log-rank test. Cox proportional hazard regression model was applied for multivariable analysis. *P*-values <0.05 were considered significant. The Stat View (SAS Institute, Cary, NC, USA) statistical package was used for all calculations.

## Results

The baseline characteristics of the 889 patients with primary MDS are shown in Table 1. Median values for the 889 patients (69% males) included: age 72 years, hemoglobin 9.6 g/dl, leucocyte count 3.4 × 10 ^9^/l, AMC 0.2 × 10^9^/l, ALC 1.2 × 10^9^/l, and platelet count 106 × 10^9^/l. Transfusion need was documented in 33% of patients and abnormal karyotype in 49%. Risk stratification by the IPSS-R was very high in 11%, high in 16%, intermediate in 21%, low in 36% and very low in 16%. The number of patients with subnormal (<0.9 × 10^9^/l), normal and above normal ALC were 261 (29%), 598 (67%) and 30 (4%), respectively; 442 (50%) and 417 (47%) patients had ALC below the median value of 1.2 × 10^9^/l and ALC (1.2–2.9) × 10^9^/l, respectively. The number of patients with subnormal AMC was 539 (61%). After a median follow-up of 27 months, 712 (80%) deaths and 116 (13%) leukemic transformations were documented. Patients with ALC above normal limits (*n*=30) were not found to be significant on the univariate analysis and were subsequently removed from further analysis and have not been represented in Table 1.

### Comparison of patients stratified by the ALC

**Figure 1:**
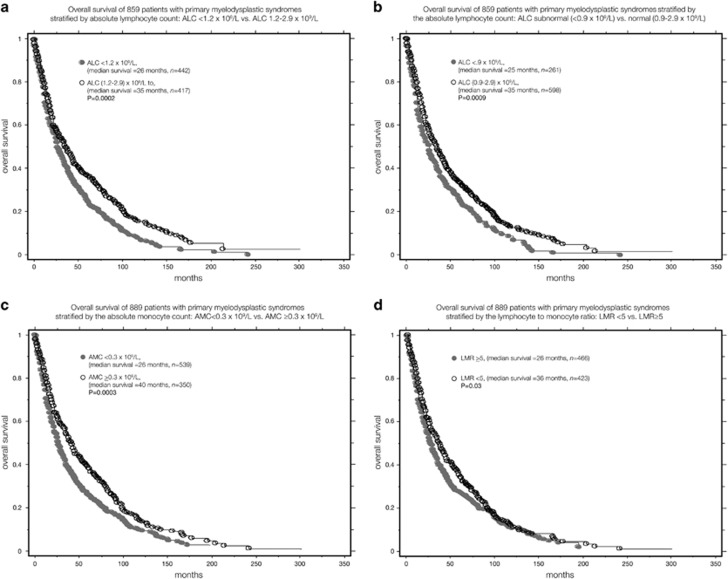
(**a**) Overall survival of 859 patients with primary MDS stratified by ALC<1.2 × 10^9^/l vs ALC 1.2–2.9 × 10^9^/l. (**b**) Overall survival of 859 patients with primary MDS stratified by the ALC subnormal (<0.9 × 10^9^/l) vs normal (0.9–2.9 × 10^9^/l). (**c**) Overall survival of 889 patients with primary MDS stratified by the AMC<0.3 × 10^9^/l vs AMC⩾0.3 × 10^9^/l. (**d**) Overall survival of 889 patients with primary MDS stratified by the LMR<5 vs LMR⩾5.

Compared with patients with normal ALC, patients with subnormal ALC clustered with several adverse disease features: lower hemoglobin (*P*=0.002), higher red blood cell transfusion need (*P*=0.0003), lower leukocyte count (*P*<0.0001), lower monocyte count (*P*=0.002) and lower platelet count (*P*<0.0001), whereas borderline association was seen with older age (*P*=0.06; Table 1). In univariate analysis, survival was adversely affected by lower ALC, treated as either a continuous variable (*P*=0.01) or a categorical variable with ALC cutoff values of <0.9 × 10^9^/l (*P*=0.001; hazard ratio (HR) 1.3, 95% confidence interval (CI) 1.1–1.5) or <1.2 × 10^9^/l (*P*=0.0002; HR 1.3, 95% CI 1.2–1.6; Table 2). Figure 1a shows Kaplan–Meier analysis for ALC <1.2 × 10^9^/l vs ALC (1.2–2.9) × 10^9^/l (median survival 26 vs 35 months, *P*=0.0002). Figure 1b shows the Kaplan–Meier analysis for ALC subnormal vs ALC normal (median survival 25 vs 35 months, *P*=0.0009). In multivariable analysis, the prognostic significance of both ALC <1.2 × 10^9^/l (*P*=0.06) and ALC <0.9 × 10^9^/l (*P*=0.1) became borderline. Other significant risk factors on both univariate and multivariable analysis are listed in Table 2. Furthermore, the inclusion of IPSS-R in the multivariate model resulted in borderline *P*-values for both ALC<1.2 × 10^9^/l (*P*=0.06) and subnormal ALC (*P*=0.1). Neither subnormal ALC (*P*=0.4) nor ALC <1.2 × 10^9^/l (*P*=0.1) affected leukemia-free survival.

### Comparison of patients stratified by the AMC and LMR

Compared with AMC⩾0.3 × 10^9^/l, monocytopenia in patients with MDS clustered with adverse disease features, such as lower hemoglobin (*P*=0.005), higher red blood cell transfusion need (*P*=0.03), lower leukocyte count (*P*<0.0001), lower platelet count (*P*<0.0001), lower absolute neutrophil count (*P*<0.0001), higher circulating (*P*=0.03) and bone marrow (*P*<0.0001) blasts, higher incidence of abnormal karyotype (*P*=0.03) and higher risk distribution in terms of both IPSS-R (*P*<0.0001) and cytogenetic risk stratification by IPSS-R (*P*=0.03; Table 1).

In univariate analysis, lower AMC was associated with inferior overall survival (*P*=0.002); significance was even more apparent when comparing patients with and without monocytopenia (*P*=0.0003; HR 1.3, 95% CI 1.1–1.5). Figure 1c. shows Kaplan–Meier analysis for AMC<0.3 × 10^9^/l vs AMC⩾0.3 × 10^9^/l (median survival 26 vs 40 months, *P*=0.0003). Similarly, there was significant association between inferior survival and LMR (*P*<0.0001) and with LMR⩾5 (*P*=0.03; HR 1.2; 95% CI 1.02–1.4). Figure 1d shows Kaplan–Meier analysis for LMR⩾5 vs LMR<5 (median survival 26 vs 36 months, *P*=0.03). In multivariable analysis, significance was retained for LMR (*P*=0.02) but was lost for LMR⩾5 (*P*=0.4) and became borderline for monocytopenia (*P*=0.09); the other risk factors used as covariates in the multivariable analysis are listed in Table 3. When IPSS-R was introduced in the multivariate model, significance was lost for monocytopenia (*P*=0.7), LMR (*P*=0.2) and LMR⩾5 (*P*=0.8).

## Discussion

ALC, as a surrogate of host immunity, has previously been associated with inferior survival in lymphomas, acute myeloid leukemia and early hematopoietic recovery following autologous stem cell transplant for myeloma.^9, 10, 11, 12^ Extensive studies in this regard were carried out in diffuse large B-cell lymphoma where investigators have found lower ALC to be an independent predictor of survival at the time of first relapse and after standard chemotherapy.^9, 16^ Subsequent studies have also suggested prognostic relevance for LMR in the infused autograft in both Hodgkin and non-Hodgkin lymphomas after autologous peripheral blood stem cell transplantation.^17, 18^

Contemporary prognostic models for primary MDS are useful but in need of further refinement, especially in light of new molecular information. The current study considers the additional prognostic role, in primary MDS, of easily accessible surrogates for host immunity. There are indeed several reports in this regard, including our previously published studies where we demonstrated an independent prognostic effect for lower ALC in both del (5q) and non-del (5q) primary MDS.^8, 14^ In the current study, we have included a much larger number of patients and also expanded our observations by including the role of monocytopenia and LMR. We show that lower ALC and AMC at the time of diagnosis clustered with adverse disease features and significantly correlated with markers for inferior outcome. We were therefore not surprised by the weaker magnitude of prognostic significance in the context of prognostic models that account for other adverse features in MDS.

The exact mechanism by which the immune system may impact prognosis in MDS is not well understood. Some studies have suggested that alterations in the dynamics and functions of T-regulatory cells could be a parameter determining disease progression and bone marrow failure in early MDS; the findings from some of these studies have suggested that the defect in T-regulatory cells in low-risk MDS favors the selection of dysplastic clones, while in high-risk group increasing number of T-regulatory cells might promote leukemic transformation.^7, 19, 20^ Regardless, our observations might provide some explanation for the therapeutic benefit of immunomodulatory agents in low-risk MDS but prospective studies are needed to examine the effect of such therapy on ALC, AMC and LMR. It is underscored, however, that some of the immunomodulatory agents might also possess direct cytotoxic activity while their effect on host immunity might be indirect.^21^

At the minimum, the findings from the current study warrant prospective monitoring of ALC and AMC during MDS clinical trials in order to determine their value as prognostic biomarkers and in providing insight into mechanism of drug action. It is possible that prognostic relevance of ALC is more or less pronounced depending on which specific lymphocyte subsets are studied and this too needs to be examined in future studies. Finally, it is important to recognize the limiting effect of multiple confounders, such as concurrent infections and use of drugs including corticosteroids, in the accurate assessment of both ALC and AMC.

## Figures and Tables

**Table 1:** Clinical and laboratory characteristics of 889 patients with myelodysplastic syndrome stratified by the absolute lymphocyte count and absolute monocyte count

*Variables*	*All patients*, n=*889*	*Patients with subnormal absolute lymphocyte count*, n=*261 (Group A)*	*Patients with absolute lymphocyte count with in the normal range*, n=*598 (Group B)*	P-*value (A vs B)*	*Patients with absolute monocyte count <0.3 × 10*^*9*^*/l*, n=*539 (Group C)*	*Patients with absolute monocyte count ⩾0.3 × 10*^*9*^*/l*, n=*350 (Group D)*	P-*value (C vs D)*
Age (years), median (range)	72 (18–98)	74 (25–94)	71 (23–98)	0.06	71 (18–98)	74 (24–95)	**<0.0001**
Gender (males), *n* (%)	616 (69)	192 (73)	409 (68)	0.1	366 (68)	250 (71)	0.3
							
*Hemoglobin g/dl, median (range)*	9.6 (5.4–15.7)	9.3 (5.8–14.6)	9.8 (5.4–15.7)	**0.002**	9.4 (5.4–15.7)	9.9 (6.2–15.7)	**0.005**
Hemoglobin<10 g/dl, *n* (%)	506 (57)	169 (65)	318 (53)	**0.002**	327 (60)	179 (51)	**0.005**
							
Transfusion needs, *n* (%)	295 (33)	111 (42)	178 (30)	**0.0003**	194 (36)	101 (29)	**0.03**
Leukocyte count × 10^9^/l, median (range)	3.4 (0.4–35)	2.4 (0.4–19.2)	3.5 (0.9–35)	**<0.0001**	2.7 (0.4–19.2)	4.8 (0.7–35)	**<0.0001**
Bone marrow blast %, median (range)	3 (0–19)	0 (0–18)	3 (0–19)	0.7	4 (0–19)	2 (0–19)	**<0.0001**
Circulating blasts %, median (range)	0 (0–18)	0 (0–18)	0 (0–18)	0.2	0 (0–18)	0 (0–18)	**0.03**
							
*Platelet count × 10*^*9*^*/l, median (range)*	106 (2–1804)	79 (2–800)	116 (7–1804)	**<0.0001**	91 (2–993)	134 (4–1804)	**<0.0001**
Platelet count<100 × 10^9^/l, *n* (%)	424 (48)	151 (57)	259 (43)	**<0.0001**	289 (54)	135 (38)	**<0.0001**
							
Absolute neutrophil count<0.8 × 10^9^/l, median (range)	237 (27)	82 (31)	151 (25)	0.06	194 (36)	43 (12)	**<0.0001**
Abnormal cytogenetics, *n* (%)	438 (49)	131 (50)	295 (49)	0.8	281 (52)	157 (45)	**0.03**
							
*IPSS-R*, n *(%)*							
Very high	97 (11)	32 (12)	62 (11)	0.1	75 (14)	22 (6)	**<0.0001**
High	141 (16)	47 (18)	90 (15)		101 (18)	40 (12)	
Intermediate	186 (21)	60 (23)	120 (20)		138 (26)	48 (14)	
Low	319 (36)	92 (35)	217 (36)		167 (31)	152 (43)	
Very low	146 (16)	30 (12)	109 (18)		58 (11)	88 (25)	
Total	889	261	598		539	350	
							
*IPSS-R cytogenetic risk group*, n *(%)*							
Very good	44 (5)	12 (5)	32 (5)	0.9	24 (4)	20 (6)	**0.03**
Good	566 (63)	165 (63)	381 (64)		328 (61)	238 (68)	
Intermediate	160 (18)	48 (18)	106 (17)		103 (19)	57 (16)	
Poor	34 (4)	11 (4)	22 (4)		20 (4)	14 (4)	
Very poor	85 (10)	25 (10)	57 (10)		64 (12)	21 (6)	
Total	889	261	598		539	350	
							
Leukemic transformation, *n* (%)	116 (13)	29 (11)	85 (14)	0.2	78 (14)	38 (10)	0.1
Deaths, *n* (%)	712 (80)	218 (83)	465 (78)		435 (81)	277 (79)	

Abbreviation: IPSS-R, Revised International Prognostic Scoring System (hemoglobin, g/dl; absolute neutrophil count, × 10^9^/l; platelets, × 10^9^/l; bone marrow blast, cytogenetic category).

Reference normal range: absolute lymphocyte count, (0.9–2.9) × 10^9^/l, absolute monocyte count (0.3–0.9) × 10^9^/l, (Mayo Clinic Laboratory).

Clinical and laboratory characteristics for patients with ALC above normal (*n*=30) have not been shown in the table. The values in bold represent the *P*-values found to be significant on analysis.

**Table 2:** Clinical and laboratory parameters adversely impacting overall survival in 889 patients with primary myelodysplastic syndromes stratified by the absolute lymphocyte count (ALC subnormal vs ALC normal; ALC<1.2 × 10^9^/l vs ALC (1.2–2.9) × 10^9^/l)

*Variables*	*Univariate analysis, HR (95% CI)* P-*value*	*Multivariate analysis* P-*value (ALC subnormal vs ALC normal)*	*Multivariate analysis* P-*value (ALC<1.2 × 10*^*9*^*/l vs ALC (1.2–2.9) × 10*^*9*^*/l)*
Older age (years)	**<0.0001**	**<0.0001**	**<0.0001**
Gender (male)	**<0.0001**	**0.01**	**0.02**
			
*Lower hemoglobin, g/dl*	**<0.0001**		
Hemoglobin <10 g/dl	**<0.0001**	**<0.0001**	**<0.0001**
			
Lower leukocyte count, × 10^9^/l	0.1		
			
*Lower platelet count, × 10*^*9*^*/l*	**<0.0001**		
Platelet count <100 × 10^9^/l	**<0.0001**	**0.004**	**0.003**
			
Absolute neutrophil count<0.8 × 10^9^/l	**0.0002**	0.3	0.3
Increased circulating blasts %	**<0.0001**	**0.003**	**0.002**
Increased bone marrow blast %	**<0.0001**	**<0.0001**	**<0.0001**
IPSS-R, cytogenetic risk group	**<0.0001**	**<0.0001**	**<0.0001**
IPSS-R, risk category	**<0.0001**		
Lower absolute lymphocyte count × 10^9^/l	**0.01**		
Absolute lymphocyte count, subnormal vs normal range	1.3 (1.1–1.5) **0.001**	0.1	
Absolute lymphocyte count <1.2 × 10^9^/l vs (1.2–2.9) × 10^9^/l	1.3 (1.2–1.6) **0.0002**		0.06

Abbreviations: ALC, absolute lymphocyte count; CI, confidence interval; HR, hazard ratio; IPSS-R, Revised International Prognostic Scoring System. Reference normal range: ALC 0.9–2.9 × 10^9^/l.

ALC <1.2 × 10^9^/l, median value of ALC in our cohort of 889 patients with primary MDS. The bold values here denote the *P*-values that were found be significant on the analysis.

**Table 3:** Clinical and laboratory parameters adversely impacting overall survival in 889 patients with primary myelodysplastic syndromes stratified by the absolute monocyte count (AMC<0.3 × 10^9^/l vs AMC ⩾0.3 × 10^9^/l) and lymphocyte-to-monocyte ratio (LMR; LMR<5 vs LMR⩾5)

*Variables*	*Univariate analysis, HR (95% CI)* P-*value*	*Multivariate analysis* P-*value, AMC<0.3 × 10*^*9*^*/l vs AMC⩾0.3 × 10*^*9*^*/l*	*Multivariate analysis* P-*value, LMR<5 vs LMR⩾5*	*Multivariate analysis, HR (95% CI)* P-*value of higher LMR*
Older age (years)	**<0.0001**	**<0.0001**	**<0.0001**	**<0.0001**
Gender (male)	**<0.0001**	**0.003**	**0.004**	**0.003**
				
*Lower hemoglobin g/dl*	**<0.0001**			
Hemoglobin <10 g/dl	**<0.0001**	**<0.0001**	**0.01**	**<0.0001**
				
Lower leukocyte count × 10^9^/l	0.05			
				
*Lower platelet count × 10*^*9*^*/l*	**<0.0001**			
Low platelet count <100 × 10^9^/l	**<0.0001**	**0.003**	**0.001**	**0.002**
				
Absolute neutrophil count <0.8 × 10^9^/l	**0.0002**	0.5	0.3	0.5
Increased circulating blasts %	**<0.0001**	**0.001**	**0.001**	**0.002**
Increased bone marrow blast %	**<0.0001**	**<0.0001**	**<0.0001**	**<0.0001**
IPSS-R, cytogenetic risk group	**<0.0001**	**<0.0001**	**<0.0001**	**<0.0001**
IPSS-R, risk category	**<0.0001**			
Lower absolute monocyte count	**0.002**			
Absolute monocyte count <0.3 × 10^9^/l vs ⩾0.3 × 10^9^/l	1.3 (1.1–1.5) **0.0003**	0.09		
Higher LMR	**<0.0001**			**0.02**
LMR<5 vs LMR⩾5	1.1 (1.0–1.3) **0.03**		0.4	

Abbreviations: AMC, absolute monocyte count; CI, confidence interval; HR, hazard ratio; IPSS-R, Revised International Prognostic Scoring System. Reference normal range: AMC (0.3–0.9) × 10^9^/l, (Mayo Clinic Laboratory). The bold values here represent the *P*-values that were found to be significant on analysis.
